# Serious neonatal morbidities are associated with differences in DNA methylation among very preterm infants

**DOI:** 10.1186/s13148-020-00942-1

**Published:** 2020-10-19

**Authors:** Todd M. Everson, T. Michael O’Shea, Amber Burt, Karen Hermetz, Brian S. Carter, Jennifer Helderman, Julie A. Hofheimer, Elisabeth C. McGowan, Charles R. Neal, Steven L. Pastyrnak, Lynne M. Smith, Antoine Soliman, Sheri A. DellaGrotta, Lynne M. Dansereau, James F. Padbury, Barry M. Lester, Carmen J. Marsit

**Affiliations:** 1 Gangarosa Department of Environmental Health, Emory University Rollins School of Public Health, Atlanta, GA USA; 2 Department of Pediatrics, University of North Carolina School of Medicine, Chapel Hill, NC USA; 3 Department of Pediatrics-Neonatology, Children’s Mercy Hospital, Kansas City, MO USA; 4 Department of Pediatrics, Wake Forest School of Medicine, Winston Salem, NC USA; 5 Department of Pediatrics, Brown Alpert Medical School and Women and Infants Hospital, Providence, RI USA; 6 Department of Pediatrics, University of Hawaii John A. Burns School of Medicine, Honolulu, HI USA; 7 Department of Pediatrics, Spectrum Health-Helen Devos Hospital, Grand Rapids, MI USA; 8 Department of Pediatrics, Lundquist Institute At Harbor-UCLA Medical Center, Torrance, CA USA; 9 Department of Pediatrics, Miller Children’s and Women’s Hospital Long Beach, Long Beach, CA USA; 10 Brown Center for the Study of Children at Risk, Brown Alpert Medical School and Women and Infants Hospital, Providence, RI USA; 11 Department of Psychiatry and Human Behavior, Brown Alpert Medical School, Providence, RI USA

**Keywords:** Neonatal, Preterm, Methylation, Epigenetics, Bronchopulmonary dysplasia, Brain injury, Infection, Retinopathy of prematurity

## Abstract

**Background:**

Infants born very preterm are more likely to experience neonatal morbidities compared to their term peers. Variations in DNA methylation (DNAm) associated with these morbidities may yield novel information about the processes impacted by these morbidities.

**Methods:**

This study included 532 infants born < 30 weeks gestation, participating in the Neonatal Neurobehavior and Outcomes in Very Preterm Infants study. We used a neonatal morbidity risk score, which was an additive index of the number of morbidities experienced during the NICU stay, including bronchopulmonary dysplasia (BPD), severe brain injury, serious neonatal infections, and severe retinopathy of prematurity. DNA was collected from buccal cells at discharge from the NICU, and DNAm was measured using the Illumina MethylationEPIC. We tested for differential methylation in association with the neonatal morbidity risk score then tested for differentially methylated regions (DMRs) and overrepresentation of biological pathways.

**Results:**

We identified ten differentially methylated CpGs (α Bonferroni-adjusted for 706,278 tests) that were associated with increasing neonatal morbidity risk scores at three intergenic regions and at *HPS4*, *SRRD*, *FGFR1OP*, *TNS3*, *TMEM266*, *LRRC3B*, *ZNF780A*, and *TENM2*. These mostly followed dose–response patterns, for 8 CpGs increasing DNAm associated with increased numbers of morbidities, while for 2 CpGs the risk score was associated with decreasing DNAm. BPD was the most substantial contributor to differential methylation. We also identified seven potential DMRs and over-representation of genes involved in Wnt signaling; however, these results were not significant after Bonferroni adjustment for multiple testing.

**Conclusions:**

Neonatal DNAm, within genes involved in fibroblast growth factor activities, cellular invasion and migration, and neuronal signaling and development, are sensitive to the neonatal health complications of prematurity. We hypothesize that these epigenetic features may be representative of an integrated marker of neonatal health and development and are promising candidates to integrate with clinical information for studying developmental impairments in childhood.

## Introduction

Much progress has been made in reducing neonatal morbidity and mortality among infants who are born very preterm (< 30 weeks of gestation). However, these neonates remain at risk for multiple serious medical complications, which often require extended stays in the neonatal intensive care unit (NICU). These include bronchopulmonary dysplasia (BPD), severe brain injury (SBI), severe retinopathy of prematurity (ROP) and serious neonatal infections (INF). Even after recovering from these potentially serious health complications and being discharged from the NICU, these infants are at increased risk of having long-term neurodevelopmental impairments.

Schmidt et al. [1] showed that BPD, SBI, and severe ROP were each independently associated with poor 18-month outcomes, including cerebral palsy, cognitive delay, hearing loss, blindness, or death; importantly, they also showed that the additive accumulation of these morbidities was a strong predictor of impairment or death. Necrotizing enterocolitis (NEC), a severe gastrointestinal infection associated with prematurity, has also been identified as a predictor of developmental impairments in childhood [2]. The inclusion of neonatal infections and NEC into a cumulative neonatal morbidity risk score, along with BPD, SBI and ROP, improves the ability to predict impairment or death by 18 months of age [3]. These severe neonatal morbidities frequently co-occur among premature infants and have some shared risk factors and pathophysiology. For instance, preterm infants with severe BPD are more likely to have severe ROP compared to infants without BPD [4], and both of these health complications may be influenced by altered regulation of angiogenic and/or angiostatic factors [5]. Additionally, severe ROP tends to occur in infants that have brain injury and impaired mental and psychomotor development [6], which may be partly due to prenatal and neonatal infections and prolonged inflammatory responses [7, 8].

We hypothesized that these neonatal morbidities may be related to underlying differences in developmental regulation or disruptions of other biological processes, which may be encoded in DNA methylation (DNAm). DNAm is an epigenetic mechanism for regulating gene expression potential that is not due to alterations in the DNA sequence, which can be heritable across cellular divisions, and typically occur at cytosine-phosphate-guanine (CpG) motifs. Infants who are born preterm have different DNAm patterns when compared to term infants [9–12] and some of these differences appear to persist into adulthood [9, 12]. Additionally, in preterm infants alterations in DNAm have been associated with sepsis [13], pain-related stress [14, 15], a potential moderator of serotonergic tone and temperament [16], and neurobehavioral responses [17]. Thus, variations in neonatal DNAm may serve as a blueprint linking preterm birth, the health complications that these infants experience, and some of the persistent developmental impairments for which they are at heightened risk.

In this study, we examined the relationships between the cumulative impact of serious neonatal morbidities experienced during NICU stay, including BPD, SBI, INF, and severe ROP, on DNAm measured at NICU discharge; we focus on this set of serious health complications because these are also risk factors for persistent impairments later in childhood [1, 3]. We hypothesize that even after these neonatal morbidities have resolved, infants likely have varying degrees of prematurity and impairment which may be reflected in their epigenetic profiles, and since prematurity has systemic effects on multiple organ systems throughout the body, this can be detected in peripheral tissues. The identification of an epigenetic signal that associates with the cumulative impact of multiple neonatal morbidities can provide unique insights into the processes that either contribute to, or are affected by, these severe health complications. We performed this analysis within the ongoing Neonatal Neurobehavior and Outcomes in Very Preterm Infants (NOVI) Study. Our long-term aims are to test whether integrating these epigenetic data with clinical data can improve the focus of interventions on infants at highest risk for persistent neurodevelopmental impairments later in childhood.

## Materials and methods

### Study population

The Neonatal Neurobehavior and Outcomes in Very Preterm Infants (NOVI) Study was conducted at nine university-affiliated NICUs from April 2014 through June 2016 who were also Vermont Oxford Network (VON) participants. All participating mothers provided written informed consent. Enrollment and consent procedures for this study were approved by local institutional review boards. Inclusion criteria included: (1) birth at < 30 weeks gestational age; (2) parental ability to read and speak English or Spanish; and (3) residence within 3 h of the NICU and follow-up clinic. Gestation estimates to determine birth < 30 weeks gestational age were based on the Extremely Low Gestational Age Newborns (ELGAN) Study criteria [18, 19]. Exclusion criteria included maternal age < 18 years, maternal cognitive impairment, infants with major congenital anomalies, maternal death, or infant death in the NICU [20, 21]. Parents of eligible infants were invited to participate in the study when infant survival was deemed likely by the attending neonatologist.

Maternal interviews were performed to collect demographic information such as age, race and ethnicity, and educational attainment, while the Hollingshead Index was used to assess socioeconomic status (SES) with a Hollingshead level V indicating low SES [22]. Infant medical records were reviewed to collect birthweight, gestational age, length of NICU stay, whether the newborn was outborn, and diagnoses of neonatal morbidities described in detail below. Outborn refers to infants that were born in a hospital without subspecialty providers of neonatal intensive care and were transferred, almost always on the day of birth, to a tertiary center for subspecialty care. Gestational age was estimated using the highest quality of available information: first using the dates of embryo retrieval or intrauterine insemination, then using fetal ultrasound, then using date of last menstrual period (LMP), and finally assigned by attending neonatologist in the absence of the above information. Postmenstrual age (PMA) is used to describe the age preterm infants during their time in the NICU and is defined as the combination of gestational age at birth plus the length of NICU stay. Buccal cells were collected for epigenomic analyses during the week of discharge from the NICU (± 3 days); thus, PMA at buccal swab collection represents the combination of gestational age at birth plus the length of NICU stay (± 3 days). Overall, 704 infants were enrolled and buccal cells were collected on 624 of these infants for epigenomic screening.

### NICU neonatal morbidities

Trained personnel at each site reviewed medical records and used Vermont-Oxford Network (VON) definitions and criteria [20] to collect information about infections, grades of ROP, and neonatal BPD through the time of discharge from the NICU. Infections included sepsis, defined as recovery of a bacterial pathogen from blood culture, and NEC, defined as having one or more of the following clinical signs: bilious gastric aspirate or emesis, abdominal distention, and occult or gross blood in stool not attributable to an anal fissure and one or more of the following radiographic signs: pneumatosis intestinalis, hepato-biliary gas, and pneumoperitoneum. Bronchopulmonary dysplasia (BPD) is one of the most common and serious forms of chronic lung disease among preterm infants, which is defined by the level of respiratory assistance required at 36 weeks PMA and can be categorized into different levels of severity (mild/moderate/severe) [23]. BPD was defined as requiring supplemental oxygen at 36 weeks PMA. For this study, any BPD regardless of severity was included in the neonatal morbidity risk score. Ascertainment of severe brain injury was based on cranial ultrasounds. When available, two ultrasound examinations were considered for each study participant; an “early” ultrasound, typically performed within a week of postnatal day 7, and a “late” ultrasound, typically performed between 36 weeks PMA and discharge. Criteria for SBI were similar to those used by Bassler et al. [3] and included parenchymal echodensity (PED), periventricular leukomalacia (PVL), and moderate to severe ventricular dilation (VDIL) with or without intraventricular hemorrhage.

Cranial ultrasounds were performed using high-frequency transducers with six standard quasi-coronal views and five para-sagittal views. Ultrasounds were read initially as part of routine clinical care and were read subsequently by a NOVI Study neuro-radiologist who classified observations according to criteria developed for the Extremely Low Gestational Age Newborn (ELGAN) study [24]. A third reading, by a NOVI Study neuro-radiologist, was performed if there was disagreement between the initial and the second readings, regarding the presence of one or more of the following: PED, PVL, or moderate to severe VDIL. Ultrasound abnormalities were classified as present if identified by at least two readers [25].

We calculated an adaptation of Bassler et al.’s validated [3] cumulative neonatal morbidity risk score by adding the number of neonatal health complications, including BPD, severe ROP, SBI, and culture-confirmed infection [3] that each infant experienced during their stay in the NICU. Due to very small numbers of infants experiencing all four morbidities, we combined those that experienced three or four morbidities into a single group, resulting in four possible levels for the risk score of 0, 1, 2, or 3 + .

### DNA methylation (DNAm) measurement, quality control, and preprocessing

DNA extraction was performed with the Isohelix Buccal Swab system (Boca Scientific). DNA was quantified with the Qubit Fluorometer (Thermo Fisher, Waltham, MA, USA), then aliquoted into standardized concentrations (~ 500 ng/uL) to allow for a total mass of 500 ng of DNA to undergo bisulfite modification and array analysis. The samples were randomly distributed across 96-well plates, rows, and chips to reduce the potential for batch effects. The Emory University Integrated Genomics Core performed bisulfite modification using the EZ DNA Methylation Kit (Zymo Research, Irvine, CA) and measured DNAm throughout the genome with the Illumina MethylationEPIC Beadarray (Illumina, San Diego, CA) following the manufacturer’s protocol. Samples with more than 5% of probes yielding detection *p* values > 1.0E−5 (74 samples), with mismatch between reported and predicted sex (7 samples), or incomplete covariate data (11 samples) were excluded. Functional normalization and beta-mixture quantile (BMIQ) normalization were performed [26], then probes on the X and Y chromosomes, those that had single nucleotide polymorphisms (SNP) within the binding region, those that could cross-hybridize to other regions of the genome [27], or probes that had low variability (range of beta-values < 0.05) [28] were excluded. After exclusions, 706,278 probes were available from 532 samples. The methylation data are publicly accessible through NCBI Gene Expression Omnibus (GEO) via accession series GSE128821.

### Estimates of tissue heterogeneity

We estimated the proportions of epithelial, fibroblast, and immune cells in our buccal samples using reference methylomes [29]. As demonstrated in our prior work, epithelial cells made up 95.7% of the cells in 95% of our samples, while immune cells made up the majority of the remaining cell types [30]. The proportions of individual immune cell subtypes were strongly inversely correlated with the proportions of epithelial cells, and very few samples had any estimated fibroblasts. Thus, we adjusted for cellular heterogeneity by including the proportions of epithelial cells as covariates in our statistical models. We also performed a sensitivity analysis to test whether the results were consistent after additionally adjusting for all immune cell proportions.

### Confounding variables

We used a directed acyclic graph (DAG) and supporting literature to demonstrate the potential confounders, cellular heterogeneity, and batch effects that needed to be adjusted for in our study (Additional file 1: Figure S1). Others have shown that the neonatal morbidities included in this risk score are more common among males [31], those who were outborn [32, 33], and those with shorter gestation [34, 35]. Similarly, differences in DNAm have been associated with sex differences [36], gestational age and preterm birth [37, 38], and outborn status may capture a number of factors that we hypothesize could contribute to differences in DNA methylation, including delays in receiving treatment and care [39]. Thus, gestational age, outborn, and sex are traditional confounders. While samples were randomized across the array to reduce bias related to batch, we additionally adjusted for a categorical batch variable to ensure that batch effects were adequately controlled for, and it is well recognized that cellular heterogeneity should be estimated and controlled for in epigenomic studies using cellular mixtures [29]. In all models, we adjusted for sex, whether the newborn was outborn (delivery occurred at a health care facility and then transferred to the NICU where enrollment in NOVI occurred), gestational age at birth (in weeks), the proportion of estimated epithelial cells, recruitment site (6-level factor), and batch (7-level factor).

### Statistical analyses: R packages

All statistical analyses were performed in R version 3.6.1. Robust linear regressions were carried out using the *MASS* package, robust standard errors were estimated using the *sandwich* package, and partial residual plots were produced with the *visreg* package. Manhattan and QQ plots were produced with the *qqman* package, and inverse-variance weighted fixed effects meta-analyses were performed with the *metafor* package.

### Statistical analyses: identification of PMA-associated CpGs

Prior to performing an epigenome-wide association study (EWAS) to identify the relationships between the neonatal morbidity risk scores and buccal cell DNAm, we explored the interrelationships between gestational age, PMA at buccal swab collection, and the risk score (Additional file 1: Figure S2). Buccal cell collection was performed close to NICU discharge and infants with more health complications typically required longer stays in the NICU. Thus, cumulative neonatal morbidity risk score and PMA at buccal swab collection are strongly correlated in our data; additionally, the number of morbidities was inversely correlated with gestational age at birth (Additional file 1: Figure S3). There is a well-recognized relationship between DNAm and aging [40], and thus, we were concerned that many of the CpGs that we identify in association with the neonatal morbidities might solely be associated with neonatal aging metrics rather than the upstream exposure of the risk score. Gestational age is likely a cause of both increasing numbers of neonatal health complications and of differential DNAm and is thus a traditional confounder that can be statistically adjusted for in our models. However, because PMA at buccal swab collection is a consequence of the exposure and cannot cause the health complications, it is not a traditional confounder and including this variable in our all of our EWAS models may result in over-adjustment [41]. To deal with this issue, we first performed an analysis aimed at identifying the CpG sites that were associated with PMA at buccal swab collection that were not driven by the antecedent neonatal morbidities. To do this, we stratified our data by level of the neonatal morbidity risk score (those with 0, 1, 2, or 3 + morbidities) and performed separate EWAS within each of these strata. DNAm was regressed on PMA at buccal swab collection, while adjusting for sex, outborn, site, batch, and epithelial cell proportions; due to the small sample size in the stratum with 3 + complications (*n* = 28), site and batch were not included in the models for this stratum. We then performed inverse-variance weighted fixed effects meta-analyses to estimate the average effect of PMA at buccal swab collection on DNAm across all levels of the neonatal morbidity risk score. Those CpGs that yielded meta-analysis *p* values < 0.05 were determined to be potential surrogate markers of PMA, independent of the neonatal health complications. For these CpGs, weeks of PMA at the time of buccal cell collection were included as an additional adjustment covariate in the EWAS for the risk score described below. CpGs that did not associate with PMA in the meta-analysis were modeled without including PMA at buccal swab collection as a covariate to avoid over-adjustment.

### Statistical analyses: epigenome-wide associations study (EWAS) for neonatal morbidity risk score

We tested for linear associations between DNAm and increasing neonatal morbidity risk scores by regressing DNAm (dependent variable) at each CpG site on the risk score as a continuous variable (range of 0–3), while adjusting for sex, whether the infant was outborn, GA at birth (weeks), recruitment site, batch (plate), and proportions of epithelial cells. Among those CpGs that were determined to be potential surrogate markers of PMA, weeks of PMA at buccal swab collection were included as an additional covariate. QQ-plots and Manhattan plots were produced to summarize the overall EWAS findings. To account for multiple testing, we implemented a false discovery rate (FDR) of 10% to determine which CpGs should be reported in the supplemental materials and Bonferroni adjustment (α = 0.05/706,278) to determine the statistically significant associations from our EWAS. We then examined the associations with risk score as 4-level factor variable to estimate the average differences in DNAm associated with each level of the risk score (1, 2, or 3 +) when compared to infants that experienced none of the neonatal morbidities. To assess whether the factor-level coefficients for differential DNAm increased in magnitude as the neonatal morbidity risk score increased, we compared the regression coefficients for 1, 2, or 3 + complications against each other via scatter plots for all CpGs that were significant at the FDR 10% threshold.

### Statistical analyses: identification of differentially methylated regions (DMRs)

We then used DMRff to search for differentially methylated regions [42]. Candidate regions were identified by specifying a maximum distance between individually differentially methylated CpGs (*maxgap*) to be 1500 bp and required a minimum *p* value (*p.cutoff*) of at least 0.001 from the EWAS. The *dmrff* function estimates regional differential methylation within these candidate regions using an extension of inverse-variance weighted meta-analysis on the parameter estimates and standard errors from the EWAS, for those CpGs within the candidate regions. Regional test-statistics as well as raw and adjusted *p* values are calculated, and the adjusted *p* values track closely with the EWAS-level Bonferroni-corrected *p* values. Due to the greedy selection through which candidate regions are identified, an FDR adjustment of the raw DMR *p* values may be inappropriate. Thus, we selected all DMRs that produced a regional *p* values less than or equal to the largest *p* value that was within the 10% FDR threshold, from the CpG-by-CpG EWAS, to report in the supplemental materials. We considered those DMRs with adjusted *p* values < 0.05 to be statistically significant.

### Statistical analyses: differential DNAm associated with individual neonatal morbidities

To examine whether individual health complications were driving the majority of the epigenetic responses at these CpGs, we then produced models without the neonatal morbidity risk score, but instead with factor variables for BPD (any versus none), SBI (any versus none), INF (any versus none), and ROP (any versus none), while adjusting for the same confounders that were included in the EWAS; again weeks of PMA at buccal swab collection was included as a covariate for CpGs that were previously identified as potential surrogate markers of PMA. For these analyses, all four morbidities were included in the same models together, to assess whether DNAm at some of these CpGs was more affected by specific health complications. We used Venn diagrams to show the relative contribution of each individual morbidities to differential methylation at these CpGs and to demonstrate the overlap in statistically significant associations for each health complication.

### Statistical analyses: over-representation of genes in biological pathways

To gain insights into the biological functions of the genes associated with the CpGs we identified, we performed over-representation analyses with methylGSA, which accounts for the number of CpGs annotated to each gene [43]. We tested for over-representation of biological pathways among the CpGs that were associated with the neonatal morbidity risk scores at an FDR of 10%.

## Results

### Characteristics of the study population

Infants in the NOVI sample for whom DNAm data and composite neonatal morbidity risk scores were available (*N* = 532), had high prevalence (*n* = 319, 60.0%) of experiencing at least one of the four neonatal morbidities (BPD, SBI, INF, or ROP), while a small subset of these infants experienced three or more of these morbidities (*n* = 28, 5.3%) (Table 1). The most common health complication in our sample was BPD (273, 51%), with severe ROP being least common (34, 6.4%). As expected, infants with increasing numbers of complications were more likely to be outborn, tended have shorter gestational ages at birth and increased PMA at buccal swab collection (*p* value < 0.001), while risk scores also differed by recruitment site (*p* value = 0.001) as well as maternal race and ethnicity (*p* value = 0.05) (Table 1).

**Table 1:** Distribution of demographic characteristics, neonatal morbidities and maternal/fetal characteristics of the study population overall and stratified by composite neonatal morbidity risk scores; ANOVA and χ^2^ tests were used to assess differences in characteristics across the levels of risk scores

Sample characteristics (*N* = 532)		Stratified by Risk Score				
	Overall	0 (*n* = 213)	1 (*n* = 193)	2 (*n* = 98)	3 + (*n* = 28)	*p* value
BPD (%)	273 (51.3)	0 (0.0)	152 (78.8)	94 (95.9)	27 (96.4)	< 0.001
INF (%)	99 (18.6)	0 (0.0)	24 (12.4)	56 (57.1)	19 (67.9)	< 0.001
SBI (%)	68 (12.8)	0 (0.0)	16 (8.3)	31 (31.6)	21 (75.0)	< 0.001
ROP (%)	34 (6.4)	0 (0.0)	1 (0.5)	15 (15.3)	18 (64.3)	< 0.001
GA (weeks)	27.01 (1.92)	27.95 (1.46)	26.92 (1.83)	25.74 (1.85)	24.91 (1.50)	< 0.001
PMA (weeks)	39.08 (3.29)	36.77 (1.87)	39.77 (2.79)	41.97 (3.31)	41.81 (2.77)	< 0.001
Race and ethnicity (%)						0.05
Hispanic other	68 (12.8)	26 (12.2)	20 (10.4)	19 (19.4)	3 (10.7)	
Hispanic white	43 (8.1)	13 (6.1)	16 (8.3)	11 (11.2)	3 (10.7)	
Non-Hispanic other	180 (33.8)	76 (35.7)	70 (36.3)	29 (29.6)	5 (17.9)	
Non-Hispanic white	234 (44.0)	94 (44.1)	86 (44.6)	39 (39.8)	15 (53.6)	
Not reported	7 (1.3)	4 (1.9)	1 (0.5)	0 (0.0)	2 (7.1)	
Outborn (%)	112 (21.1)	30 (14.1)	34 (17.6)	37 (37.8)	11 (39.3)	< 0.001
Male (%)	297 (55.8)	130 (61.0)	100 (51.8)	54 (55.1)	13 (46.4)	0.201
Maternal age (years)	29.10 (6.39)	29.13 (6.60)	29.49 (6.02)	28.35 (6.77)	28.95 (5.93)	0.556
Education < HS/GED (%)	72 (13.9)	28 (13.7)	21 (11.1)	19 (19.6)	4 (14.8)	0.276
Lowest SES (%)	42 (8.1)	15 (7.4)	15 (7.9)	8 (8.2)	4 (14.8)	0.615
Recruitment site (%)						0.001
WIH	94 (17.7)	30 (14.1)	47 (24.4)	12 (12.2)	5 (17.9)	
SHD	100 (18.8)	52 (24.4)	31 (16.1)	12 (12.2)	5 (17.9)	
KMC	88 (16.5)	44 (20.7)	25 (13.0)	15 (15.3)	4 (14.3)	
CMH	68 (12.8)	16 (7.5)	25 (13.0)	22 (22.4)	5 (17.9)	
WFU	113 (21.2)	50 (23.5)	35 (18.1)	21 (21.4)	7 (25.0)	
LUB	69 (13.0)	21 (9.9)	30 (15.5)	16 (16.3)	2 (7.1)	

*BPD* bronchopulmonary dysplasia, *INF* neonatal infection, *SBI* severe brain injury, *ROP* severe retinopathy of prematurity, *GA* gestational age, *PMA* postmenstrual age, *HS* high school, *GED* general educational development test, *SES* socioeconomic status

### Identification of PMA-associated CpGs

Infants with more health complications tended to have longer stays at the NICU and thus had increased PMA at the time of buccal swab collection and age metrics can have strong associations with DNAm. Thus, we aimed to limit the chances of us detecting CpGs that were surrogate markers of PMA at the time of buccal swab collection when we performed an EWAS of the neonatal morbidity risk score. To do this, we first performed an EWAS of PMA at buccal cell collection to identify those CpGs that are associated with aging, independent of the risk scores. We identified a very substantial epigenetic signal for weeks of PMA at buccal swab collection with 13,034, 85,837, and 142,580 CpGs being associated with PMA at various thresholds of statistical significance: Bonferroni correction, FDR of 10%, and raw *p* values < 0.05, respectively (Additional file 1^1^ Figure S4). For the 142,580 CpGs yielding even modest evidence of an association with PMA, we chose to include PMA in the EWAS models of neonatal morbidity risk scores. On the other hand, we were not concerned about DNAm being a potential surrogate marker of PMA at buccal cell collection for the other 563,698 CpGs, and thus, PMA at buccal swab collection was not included as a covariate for those CpGs in the EWAS.

### Epigenome-wide associations study (EWAS) for composite neonatal morbidity risk score

**Fig. 1:**
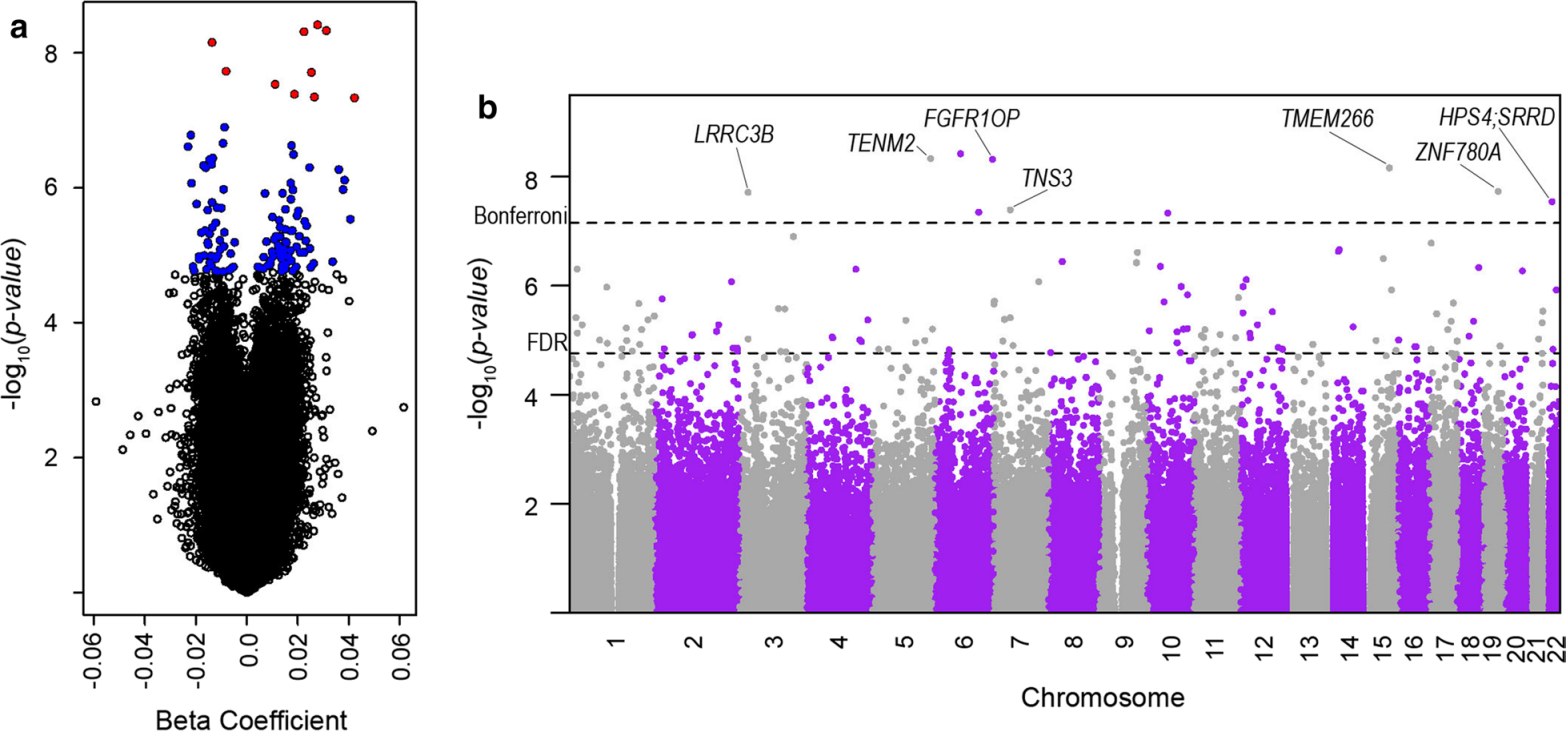
Volcano plot **a** of the beta coefficients and − log_10_(*p* values) from the epigenome-wide association study (EWAS) (blue = FDR < 10%; red = Bonferroni), and a Manhattan plot **b** of the genomic distribution of these results with gene names annotated to those CpGs that passed the Bonferroni threshold

We then performed an EWAS to identify the average differences in DNAm for the cumulative increase in the number of health complications that the infants experienced in the NICU; all models were adjusted for sex, gestational age, outborn, batch, and proportions of epithelial cells, while PMA (weeks) at buccal collection was adjusted for the CpGs described above. We identified ten CpGs that were differentially methylated in association with the neonatal morbidity risk scores after Bonferroni adjustment (Table 2) which will be referred to as the genome-wide significant CpGs from this point forward. Additionally, 125 CpGs produced associations within a 10% FDR (Fig. 1 and Additional file 1: Figure S5 and Additional file 2: Excel Table E1); these 125 CpGs were included in all subsequent analyses and all results for these CpGs are reported in the supplemental materials. Among the genome-wide significant results, three CpGs were intergenic including the most statistically significant association (cg09787236 on 6q13; *p* value = 3.82E−09), while the other seven CpGs were annotated to *HPS4, SRRD*, *FGFR1OP*, *TNS3*, *TMEM266*, *LRRC3B*, *ZNF780A* and *TENM2*. The only genome-wide significant CpG that was also identified as a potential marker of PMA was cg26838315 (10q21.1). This CpG exhibited the largest magnitude of effect for an increase in the risk score (*β*_1_ = 0.042; *p* value = 4.64E−08), which can be interpreted as an average increase in DNAm of 4.2% for each increase in the number of neonatal morbidities, after adjusting for confounders and PMA at buccal swab collection. Interestingly, PMA was associated with lower levels of DNAm at this CpG (*β*1 = − 0.007; *p* value = 0.00023). Thus, DNAm at cg26838315 is lower with neonatal aging, while having more neonatal morbidities, which results in an extended length of stay in the NICU, was associated with increasing levels of DNAm. We performed a sensitivity analyses to examine whether additional adjustment for immune cell proportions attenuated our findings for these 10 CpGs and found that these additional adjustments had no impact on our findings (Additional file 2: Excel Table E2).

**Table 2:** CpG-specific associations between DNAm with a linear increase in composite neonatal morbidity risk score, adjusted for gestational age at birth (weeks), sex, whether the infant was outborn, the proportion of epithelial cells, recruitment site, batch, and PMA (weeks) where appropriate; the beta coefficient represents the average difference in DNA methylation for each increase in the number of morbidities that the infant experienced (BPD, SBI, ROP, or infection); gene annotations verified in UCSC Genome Browser (hg19)

CpG Site	Coef	Std. Err	*p* value	FDR	Chr	Pos	Gene	Region
cg06343740	0.0111	0.0020	2.91E−08	0.0029	chr22	26878410	*HPS4;SRRD*	5′UTR;TSS1500;Body
cg07846767	0.0224	0.0038	4.83E−09	0.0011	chr6	167443722	*FGFR1OP*	Body
cg08303167	0.0265	0.0048	4.50E−08	0.0033	chr6	126953300	6q22.33	–
cg09787236	0.0278	0.0047	3.82E−09	0.0011	chr6	74652865	6q13	–
cg11255857	0.0187	0.0034	4.08E−08	0.0033	chr7	47431773	*TNS3*	Body
cg12861771	− 0.0137	0.0024	6.96E−09	0.0012	chr15	76442477	*TMEM266*	Body
cg16636226	0.0254	0.0045	1.93E−08	0.0023	chr3	26669219	*LRRC3B*	5′UTR
cg20938154	− 0.0081	0.0014	1.86E−08	0.0023	chr19	40597336	*ZNF780A*	TSS1500
cg24517837	0.0312	0.0053	4.65E−09	0.0011	chr5	167391645	*TENM2*	Body
cg26838315	0.0423	0.0077	4.64E−08	0.0033	chr10	59559554	10q21.1	–

*CpG* cytosine-phosphate-guanine site, *Coef.* estimated difference in DNAm associated with an increase of one in the neonatal morbidity risk score, *Std. Err.* standard error, *FDR* false discovery rate, *Chr.* chromosome, *Pos.* genomic location (hg19)

**Fig. 2:**
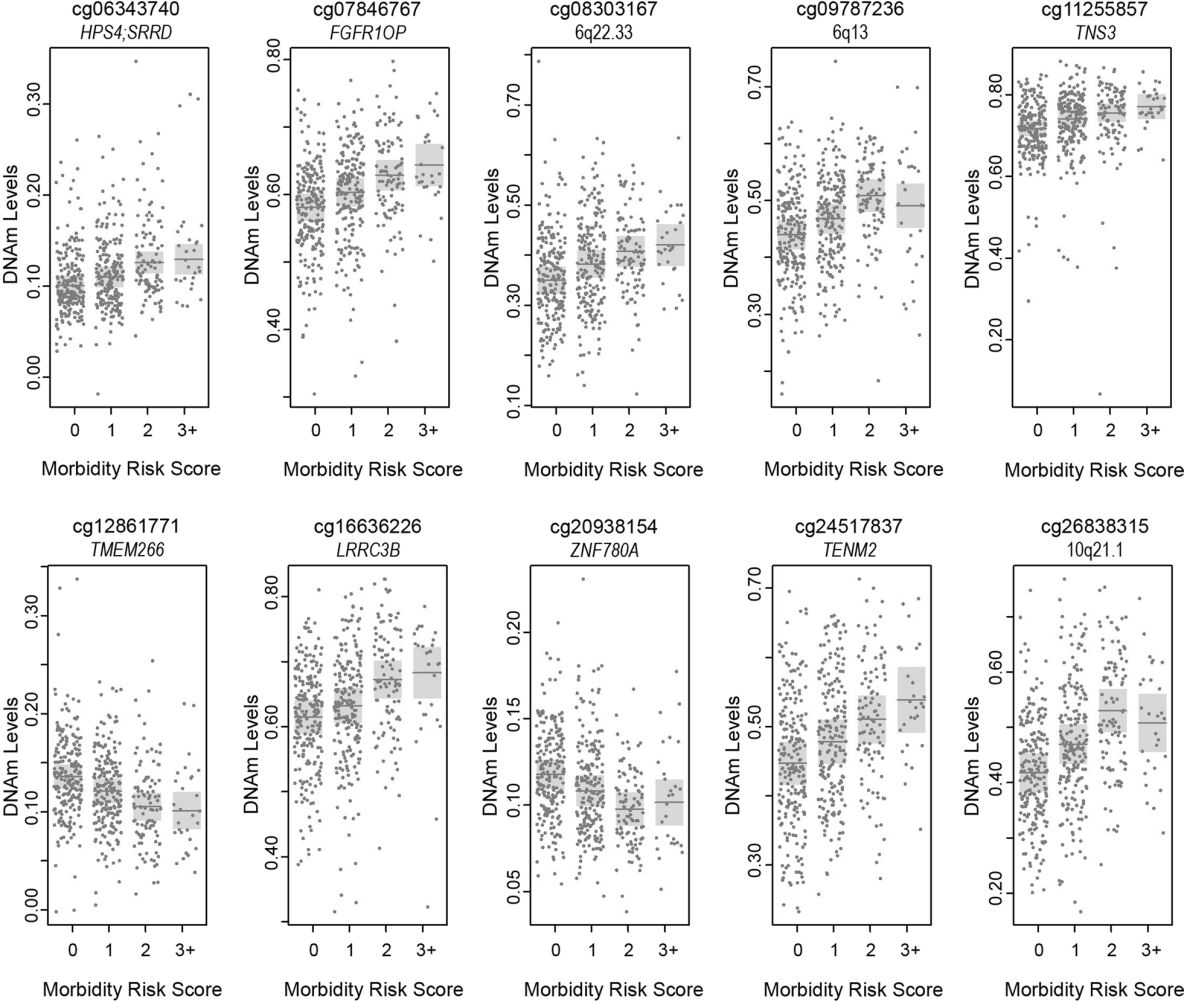
Partial residual plots of the estimated average DNAm levels (dark grey lines) within each level of the neonatal morbidity risk score, adjusted for sex, gestational age at birth, outborn, recruitment site, batch, proportions of epithelial cells, and PMA (weeks) were appropriate, for the top 10 CpGs from the EWAS

We then performed secondary analyses to assess whether the relationship between DNAm and the neonatal morbidity risk scores was in fact linear or following a dose–response pattern. Thus, we characterized the average differential methylation associated with each level of the risk score versus those infants that did not experience any of BPD, SBI, INF, or ROP, again using linear models with the same adjustment for covariates but this time including the risk score as a 4-level factor with a score of zero as the referent group (Additional file 2: Excel Table E3). We used scatter plots of the magnitudes of differential methylation (reference score = 0) at each CpG to show that a risk score of 2 produced a greater amount of differential DNAm, compared to a risk score of 1, for almost all CpGs; and for most CpGs (72%), a risk score of 3 + produced greater differential methylation than the risk score of 2 (Additional file 1: Figure S6). Thus, the majority of the signal that we observed was predominantly driven by a dose response relationship and not heavily driven by a single level of the neonatal morbidity risk scores. These dose–response patterns were also apparent among the ten genome-wide significant CpGs, although for two of these CpGs (cg09787236 and cg26838316) the highest level of the risk score (3 +) did not fit this pattern (Fig. 2).

### Identification of differentially methylated regions (DMRs)

We then tested for differentially methylated regions (DMRs) using DMRff, which performs a modified meta-analysis on the EWAS parameter estimates for differential methylation at the CpGs within candidate regions that are defined by genomic proximity [42]. We allowed for a maximum distance of 1500 bp between individually differentially methylated CpGs. Only those individual CpG sites from the EWAS with a *p* value < 0.001 were considered differentially methylated. Using these criteria, 1744 candidate regions were present and 25 DMRs with at least two differentially methylated CpGs were identified. None of these DMRs exhibited significant regional differential methylation at the Bonferroni-adjusted threshold; seven DMRs did produce regional *p* values that were equal to or less than the largest *p* value from the EWAS that was within an FDR 10% (Table 3).

**Table 3:** Differentially methylated regions (DMRs) containing at least two CpGs, with an estimated regional change in DNAm of at least 1% for each increase in the neonatal morbidity risk score and yielding a DMR *p* value < 0.0001

DMR	# CpGs	Coef	Std. Err	*p* value	Gene	Region
chr2:127822822–127823595	2	− 0.0098	0.0019	2.30E−07	*BIN1*	Body
chr20:44636682–44636831	2	0.0119	0.0024	3.86E−07	*MMP9*	TSS1500
chr12:4489056–4489155	3	0.0145	0.0029	4.16E−07	*FGF23*	TSS200;TSS1500
chr8:70360215–70361190	5	0.0077	0.0015	5.69E−07	*LINC01603*	Body;TSS200;TSS1500
chr13:95358540–95358924	2	− 0.0140	0.0029	1.89E−06	*–*	–
chr10:8094142–8094482	3	0.0107	0.0024	1.05E−05	*FLJ45983*	Body
chr3:142956052–142956059	2	0.0091	0.0021	1.55E−05	*–*	–

*DMR* differentially methylated region, *Coef.* estimated difference in DNAm associated with an increase of one in the neonatal morbidity risk score, *Std. Err.* standard error

### Differential DNAm associated with individual neonatal morbidities

**Fig. 3:**
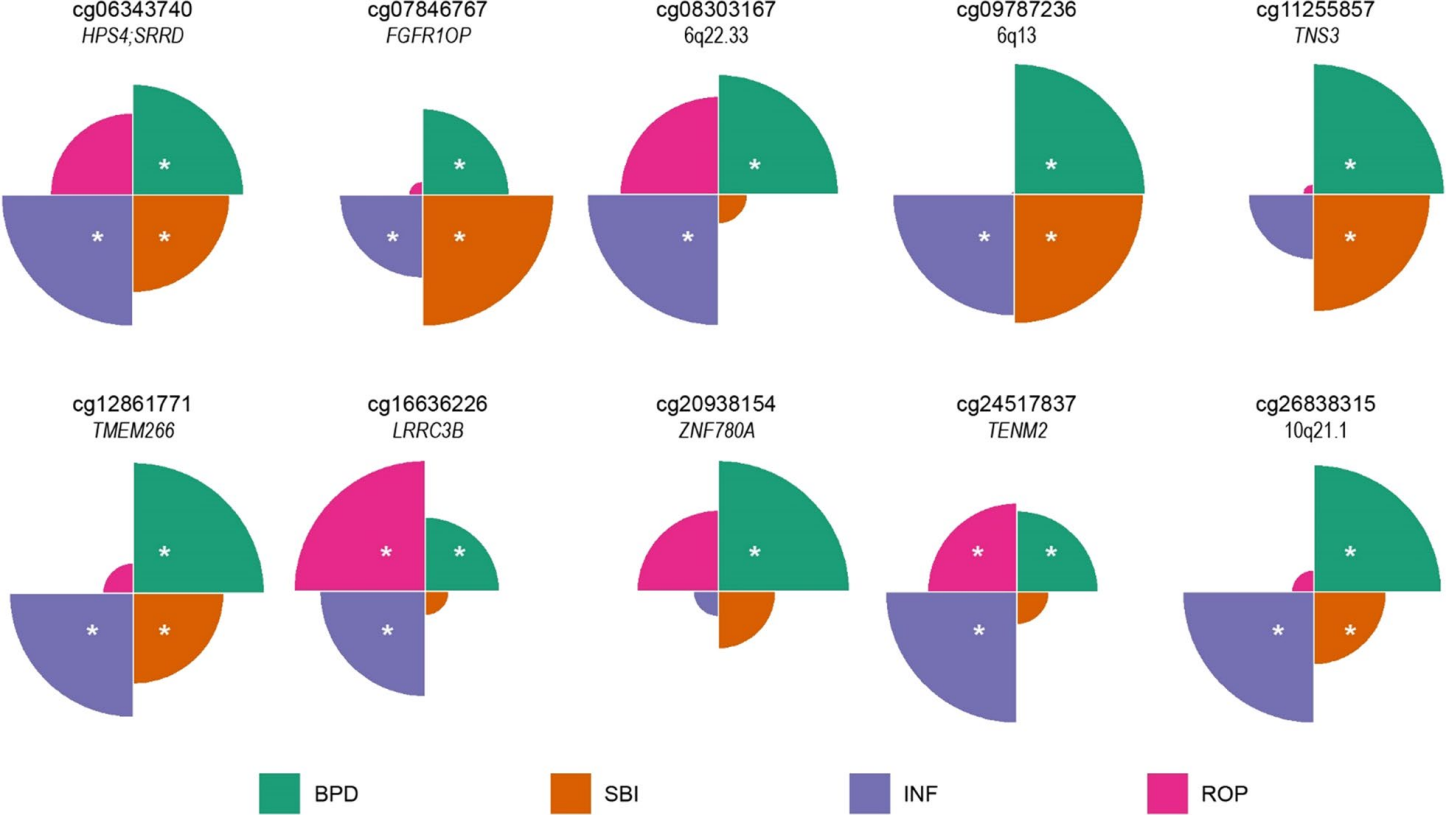
Circular bar plots of the estimated average differences in DNAm levels associated with BPD (green), SBI (orange), INF (blue), and ROP (pink), adjusted for sex, gestational age at birth, outborn, recruitment site, batch, proportions of epithelial cells, and PMA (weeks) where appropriate, for the top 10 CpGs from the EWAS; all four morbidities were included in the models, and thus, the size of each quadrant represents the relative magnitude of the difference in DNAm for each morbidity; morbidities that were differentially methylated (p-values < 0.05) while adjusting for all other morbidities are indicated with an asterisk (*)

We then evaluated whether the observed associations between DNAm and the cumulative neonatal morbidity risk scores were being driven by any individual health complication. We regressed DNAm on dichotomous indicator variables for BPD, SBI, INF, and ROP (all four included in the model), while adjusting for the same confounding variables that were included in the EWAS (Additional file 2: Excel Table E4). We found that DNAm levels at almost all CpGs were associated (*p* value < 0.05) with BPD (89.6%), followed by SBI (45.6%), INF (44.8%), and ROP (20.0%). Additionally, 23 CpGs were associated solely with BPD when all four complications were in the model, while only five, one, and zero CpGs were solely associated with INF, SBI, and ROP, respectively (Additional file 1: Figure S7). We also found that levels of differential methylation associated with each of the health complications were highly correlated with the level of differential methylation associated with an increase in one for the neonatal morbidity risk score (Additional file 1: Figure S8). These same patterns of association for the individual health complications were also captured by the ten genome-wide significant CpGs from our EWAS (Table 4 and Fig. 3), with BPD appearing to be the predominant driver of the epigenetic signal from the risk score. However, it is also important to point out that SBI, INF, and/or ROP explained differences in DNAm at many of these CpGs, including all genome-wide significant CpGs, even when BPD was included in the model. Thus, the differences in DNAm that we detected are not merely capturing the effects of BPD, but instead may be reflecting a more integrated marker of neonatal health.

**Table 4:** CpG-specific associations between DNAm with BPD, SBI, INF and ROP (all were included in the model) while adjusting for gestational age at birth (weeks), sex, whether the infant was outborn, the proportion of epithelial cells, recruitment site, batch, and PMA (weeks) where appropriate; the beta coefficient represents the average difference in DNA methylation for infants that experienced that morbidity in the NICU, relative to those that did not, while adjusting for the other health complications included in the neonatal morbidity risk score

CpG	Gene	BPD		SBI		INF		ROP	
		Coef	*p* value	Coef	*p* value	Coef	*p* value	Coef	*p* value
cg06343740	*HPS4;SRRD*	0.0111	2.30E−03	0.0098	4.01E−02	0.0131	4.81E−03	0.0083	3.91E−01
cg07846767	*FGFR1OP*	0.0228	1.03E−03	0.0346	9.64E−05	0.0218	1.03E−02	0.0037	7.88E−01
cg08303167	6q22.33	0.0320	9.73E−04	0.0078	4.96E−01	0.0349	1.24E−03	0.0261	9.39E−02
cg09787236	6q13	0.0320	1.41E−04	0.0315	6.90E−03	0.0296	1.99E−03	0.0012	9.57E−01
cg11255857	*TNS3*	0.0257	4.15E−05	0.0229	4.97E−03	0.0127	1.08E−01	− 0.0021	8.89E−01
cg12861771	*TMEM266*	− 0.0171	3.26E−05	− 0.0118	4.22E−02	− 0.0162	4.89E−04	0.0040	7.33E−01
cg16636226	*LRRC3B*	0.0235	4.06E−03	0.0076	5.11E−01	0.0332	2.50E−04	0.0414	1.62E−03
cg20938154	*ZNF780A*	− 0.0130	9.11E−07	− 0.0057	9.00E−02	− 0.0025	4.37E−01	− 0.0081	8.00E−02
cg24517837	*TENM2*	0.0302	2.97E−03	0.0120	3.25E−01	0.0486	3.11E−05	0.0331	4.73E−02
cg26838315	10q21.1	0.0532	3.00E−04	0.0303	3.47E−02	0.0547	1.69E−04	0.0093	6.69E−01

*CpG* cytosine-phosphate-guanine site, *Coef.* estimated difference in DNAm associated with the presence of each neonatal morbidities, *BPD* Bronchopulmonary dysplasia, *INF* neonatal infection, *SBI* serious brain injury, *ROP* severe retinopathy of prematurity

### Over-representation of genes in biological pathways

We explored the biological activities of the gene sets that were most strongly associated with the neonatal morbidity risk scores. None of the identified pathways were significantly enriched after adjustment for multiple testing and only three pathways were enriched at a significance level of *p* value < 0.10, including Cushing syndrome (hsa:04934), Wnt signaling pathway (hsa:04310), and Oxytocin signaling pathway (hsa:04921). We considered that our gene set, which only included 125 CpGs, may be underpowered to detect enrichment and thus relaxed the significance threshold to FDR levels of 15% (243 CpGs), 20% (375 CpGs), and 25% (549 CpGs) and then reran the enrichment analyses. Only one pathway was nominally enriched for (*p* value < 0.05) in all three of these analyses, the Wnt signaling pathway (hsa:04,310) from which our CpGs included 7 genes (*p* value = 0.049), 9 genes (*p* value = 0.0032), and 11 genes (*p* value = 0.0049) for the FDR cutoffs of 15%, 20%, and 25%, respectively. However, again, none of these *p* values were statistically significant after adjusting for multiple testing.

### Summary of results

In summary, cumulative neonatal morbidities were strongly associated with increasing DNAm at eight CpGs and decreasing DNAm at two CpGs (α adjusted for 706,278 tests). These relationships were largely linear and followed dose–response patterns, demonstrating the robustness of these associations. Additionally, nine of these ten CpGs were associated with more than one neonatal health complication, when all complications were included as dichotomous variables within the same model. Thus, the differences in DNAm that we detected were not being driven by any single complication, but instead may represent a more integrated marker of prematurity or neonatal health.

## Discussion

In a cohort of infants that were born very preterm (< 30 weeks gestation), we identified a DNAm signature associated with increasing numbers of neonatal morbidities at specific CpG sites in three intergenic regions on 6q22.33, 6q13, and 10q21.1, and within the genes, *HPS4*, *SRRD*, *FGFR1OP*, *TNS3*, *TMEM266*, *LRRC3B*, *ZNF780A*, and *TENM2*. Additionally, we observed evidence of regional differences in DNAm at *BIN1*, *MMP9*, *FGF23*, *LINC01603*, *FLJ45983* and two intergenic regions, although these regional differences did not meet the Bonferroni-adjusted significance threshold. Among the four complications included in the neonatal morbidity risk score, BPD appeared to be the primary driver of the epigenetic responses. However, SBI, INF, and/or ROP were associated with differential methylation at all of the genome-wide significant CpG sites in addition to BPD. Thus, these epigenetic differences may be related to common processes that either contribute to, or are affected by, these severe neonatal health complications.

Among these 13 genes, some common biological processes were represented. For instance two CpG sites were within genes, *TNS3* and *FGFR1OP*, that can interact with and potentially influence the function of the fibroblast growth factor receptor (*FGFR*), and which has previously been observed in cases of developmental disorder and myeloproliferative disorder, respectively [44, 45]. One DMR was within *FGF23*, which has pleiotropic effects on multiple processes, including metabolism of phosphate, calcium and sodium, bone mineralization [46, 47]. *FGF23* does appear to be highly expressed in the neonatal period [48, 49], and as a family, FGFs have extensive roles during embryonic development in processes related to organogenesis, such as proliferation and differentiation [50]. Additionally, FGFs are significant angiogenic factors [51, 52] and dysregulation of angiogenic processes are thought to contribute to the development of some of these neonatal health complications [5]. *LRRC3B* and *MMP9* have been associated with mechanisms of cellular proliferation, invasion, and cell cycle regulation [53, 54], and interestingly, *LRRC3B* has been shown to inhibit the expression of *MMP9* in one study [55]. *TMEM266* produces a functional voltage sensor at synapses in the cerebellum and thus may play important roles of neuronal signaling [56, 57] while *TENM2* appears to be involved in neuronal growth and migration [58]. *BIN1* has been associated with neurodegenerative diseases [59] and may be involved in neuronal function [60]. The potential roles that these genes play in the neonatal period are not well described, and it is not clear if buccal cell DNAm patterns at these genes are reflective of DNAm patterns at these same genes in neuronal, respiratory, and immune cells. However, our data suggest that cellular process related to fibroblast growth factor activities, cellular proliferation and invasion, and neural development and signaling may be sensitive to severe neonatal morbidities in infants that are born very preterm.

While our study focused on epigenetic variation that is associated with the burden of multiple neonatal morbidities in very preterm infants, other studies have previously shown that preterm birth itself is associated with differential DNAm in several different tissues, including placenta [61], neonatal blood [9–11], and neonatal saliva [37]. Many of the CpG sites that have been associated with preterm birth in these prior studies are within genes that are involved in neuronal development and/or neurodegenerative disorders, and we have previously found that neonatal neurobehavioral responses in preterm infants are associated with differences in DNAm [30]. Furthermore, some of the CpGs associated with preterm birth have been shown to be differentially methylated in adult blood, suggesting that DNAm could potentially mediate some of the long-term health consequences that are associated with prematurity [9, 12]. Our findings provide supporting evidence that neonatal DNAm in preterm infants may inform future health outcomes by identifying epigenetic variation associated with neonatal morbidities, BPD, SBI, INF, and ROP, which are predictive of persistent developmental impairments [1, 3].

In summary, we identified a robust epigenetic signature of neonatal health complications at a genome-wide significance threshold that exhibited dose response relationships with the neonatal morbidity risk score and occurred within genes whose epigenetic dysregulation could plausibly be related to future developmental outcomes. However, we also want to stress some of the limitations of this analysis, including potential residual confounding, lack of independent replication and lack of access to ideal target tissues. There is likely measurement error for some of our different age metrics (gestational age and PMA), since the date of conception was estimated using the best information available, but gold standard assessments were not available for many of our newborns; thus, there may be some residual confounding from these age metrics. Due to the uniqueness of our sample of very preterm infants (< 30 weeks of gestation), we are not aware of an independent population with neonatal buccal cell DNAm data within which to perform a replications analysis. Additionally, although DNAm was measured in a peripheral tissue, target tissues related to these health complications are mostly inaccessible for observational studies of living children. Buccal cells, on the other hand, are one of the most accessible tissues for studies of children [62] and have been suggested to be one of the preferred surrogate tissues for epigenetic studies of psychiatric and neurobehavioral outcomes [63–65]. It is possible that the observed differences in DNAm could be correlated with relevant biological process affected by prematurity or represent surrogate indicator of prematurity and thus may be predictive of future health consequences even if they are not causally involved in those pathological processes. Additionally, we used a cumulative risk index to assess the impact of multiple neonatal morbidities, rather than performing separate EWAS analyses of each morbidity independently. This approach reduced the multiple testing burden that would have been necessary for testing each morbidity separately. However, by focusing on this risk index, we likely did not capture all of the epigenetic responses that may be related to these specific morbidities. Future studies could test for differential DNAm associated specifically with SBI, BPD, ROP, and neonatal infections, or with other impairments that were not included in our analyses. Despite these limitations, our results provide evidence regarding the impact of neonatal morbidities among very preterm infants on the early-life epigenome and provide promising candidates for future studies of epigenetic predictors of neurodevelopmental impairment.

We observed differences of 1–4% methylation, for each unit increase in morbidity risk score, and when comparing those with 3 + morbidities to those with no morbidities, the largest differences were around 9% (at cg26838315 and cg24517837). These effect sizes, while small on the absolute scale, are consistent with what is observed in other epidemiologic studies of DNA methylation and children’s health [66]. We should be cautious in interpreting small differences in DNA methylation and the potential functional consequences of those differences. However, it is important to emphasize that our study, and most epidemiologic studies, measures DNA methylation from tissue samples that are composed of many cells. Thus, for a given CpG site, the beta-value (percent DNAm) represents the proportion of alleles, across many cells, where that CpG was methylated. However, within an individual cell, a CpG is either methylated or unmethylated. Thus, a “small” difference in percent DNAm at a particular CpG is likely representative of a subpopulation of cells with a different methylation state at that genomic location. Because DNAm can be inherited across cellular divisions, these differences may be representative of prior exposures or events and they may persist with growth and aging. Important future research should investigate whether these differences in DNAm persist, amplify, or attenuate as these children age, and whether these dynamics are related to differences in neurodevelopmental impairments. We aim to investigate such DNAm dynamics in relation to these outcomes with future studies in this ongoing longitudinal cohort of babies that were born very preterm.

## Conclusions

In summary, the serious neonatal morbidities experienced by infants that are born very preterm appear to leave a signature on the epigenome at the time that those infants are discharged from the NICU. While BPD was the most substantial driver of this epigenetic signature, all of these health complications of prematurity contributed to the observed differential methylation. Additionally, these epigenetic variations occur within genes involved in developmental processes. Thus, we hypothesize that these epigenetic features may be representative of an integrated marker of neonatal health and development and should be considered as potential candidates for future studies of developmental impairments in children who were born premature. Our ongoing work in NOVI aims to examine whether these differences in DNAm persist with aging and to integrate these epigenetic data with clinical information to test whether the inclusion of epigenetic information can improve predictions about neurodevelopmental impairments in childhood.

## Supplementary information

**Additional file 1:** Supplemental Figures S1–S8.

**Additional file 2:** Supplemental Excel Tables 1–4.

## Data Availability

The raw and processed DNAm data are publicly accessible through NCBI Gene Expression Omnibus (GEO) via accession series GSE128821.

## Abbreviations

BPD: Bronchopulmonary dysplasia
DMR: Differentially methylated regions
DNAm: DNA methylation
INF: Serious neonatal infections
NEC: Necrotizing enterocolitis
NICU: Neonatal intensive care unit
NOVI: Neonatal Neurobehavior and Outcomes in Very Preterm Infants Study
ROP: Severe retinopathy of prematurity
SBI: Severe brain injury
SBC: Skin & Blood clock
PBC: PedBE clock
PMA: Postmenstrual age
