# Supplementary material for: Serious neonatal morbidities are associated with differences in DNA methylation among very preterm infants
Source: Clin Epigenetics. 2020 Oct 19;12:151. doi: 10.1186/s13148-020-00942-1 (PMC7574188; doi:10.1186/s13148-020-00942-1)

**Supplemental Figure S1:** Directed acyclic graph (DAG) demonstrating the expected interrelationships between cumulative neonatal morbidities (including bronchopulmonary dysplasia (BPD), severe brain injury (SBI), infection (INF), and severe retinopathy of prematurity (ROP)) the outcome variable (DNA methylation, measured at discharge from the NICU), potential confounders (sex, outborn, shorter gestation, recruitment site), batch variables, cellular heterogeneity, and postmenstrual age (PMA) at the time of buccal swab collection. Variables that were adjusted for within our EWAS models have boxes around them. PMA was not adjusted for in all EWAS models, since it may result in over-adjustment, but was included for those CpGs that we identified as being associated with PMA independent of these medical morbidities.


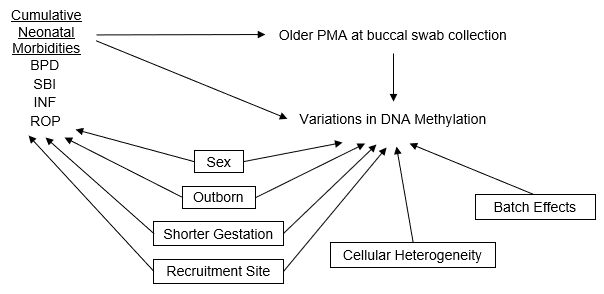


**Supplemental Figure S2:** Histogram**s** showing the distributions of gestational age, PMA at discharge from the NICU, and a scatter plot of the relationships between gestational age and PMA; infants with a risk score of 0 = black, a risk score of 1 = blue, a risk score of 2 = green, and a risk score of 3+ = red.

**
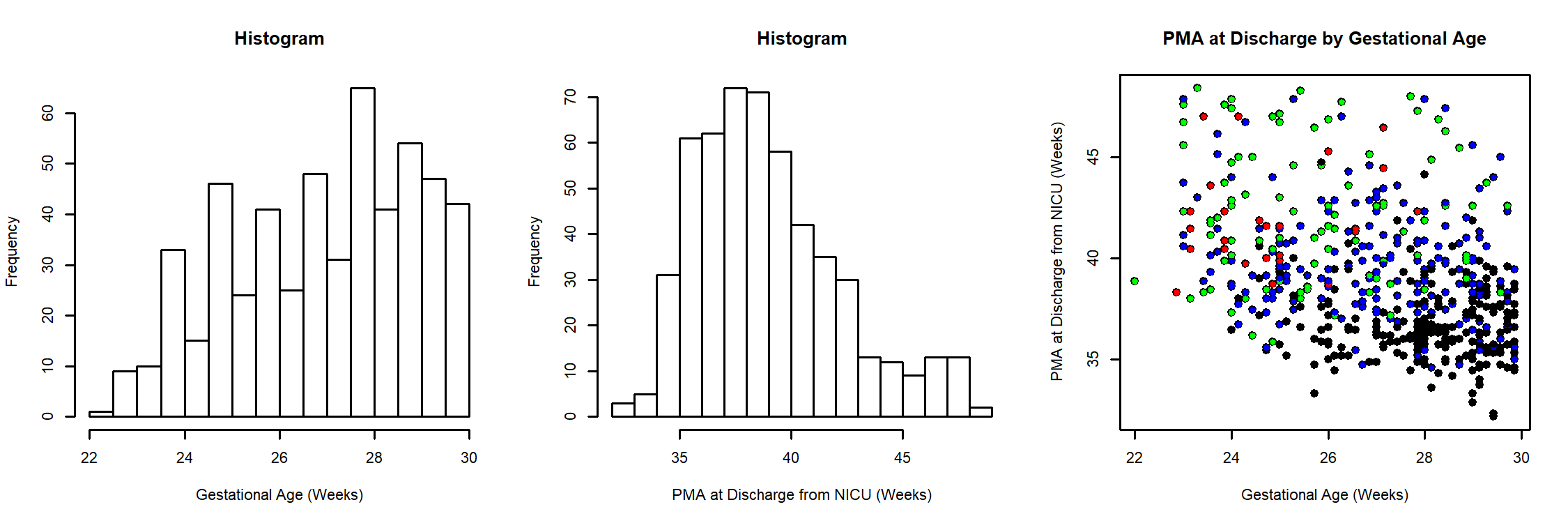
**

**Supplemental Figure S3:** Increasing numbers of health complications was a cause of longer stay at the NICU, and infants with more complications were had increased PMA at the time that buccal swabs were collected for epigenomic analyses (Pearson’s correlation coefficient = 0.60, p-value < 2.20E-16); additionally, infants born with shorter gestational age were more likely to develop multiple health complications (Pearson’s correlation coefficient = -0.48, p-value < 2.20E-16).


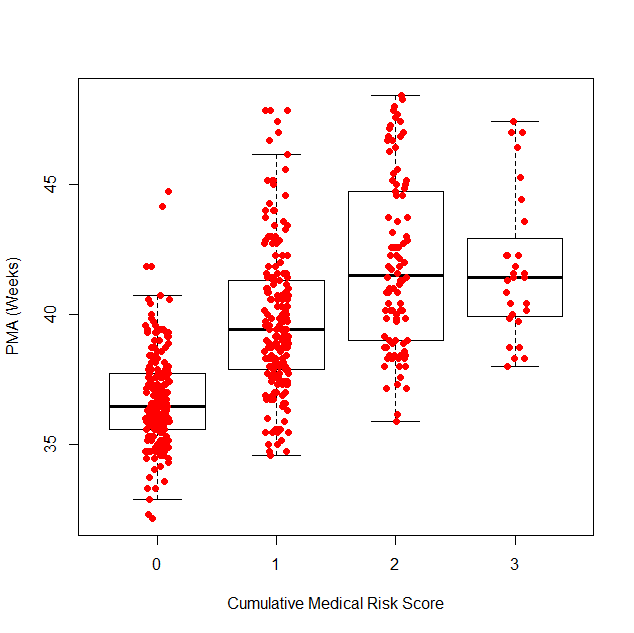

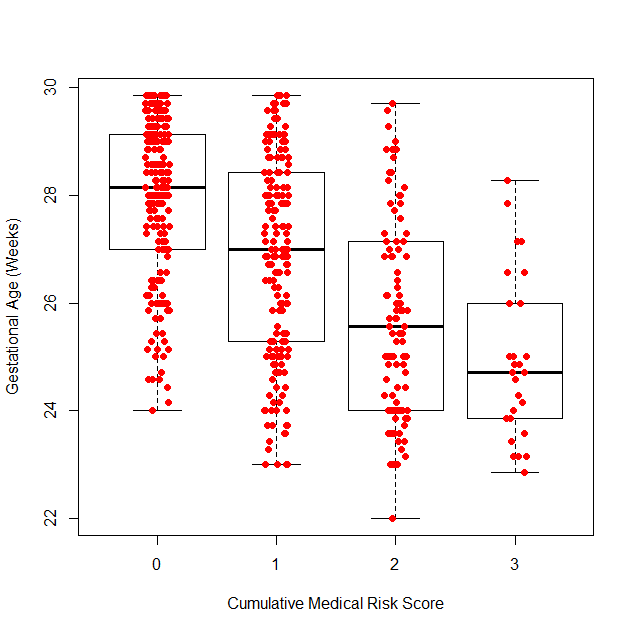


**Supplemental Figure S4:** Volcano plot of the meta-analysis results to identify the PMA-associated CpGs; blue represents those associations within a 10% FDR threshold and red represents those associations within a Bonferroni-adjusted threshold for statistical significance.


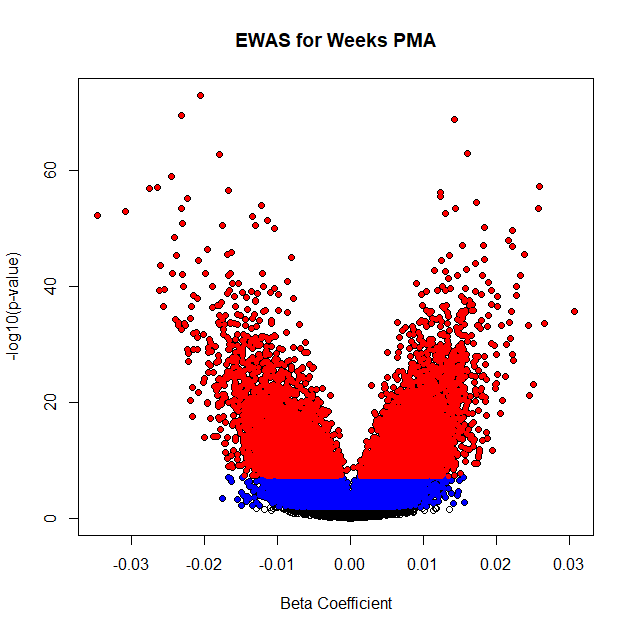


**Supplemental Figure S5:** QQ-plot of the EWAS identifying CpG-specific differential methylation associated with increasing levels of the neonatal morbidity risk scores.


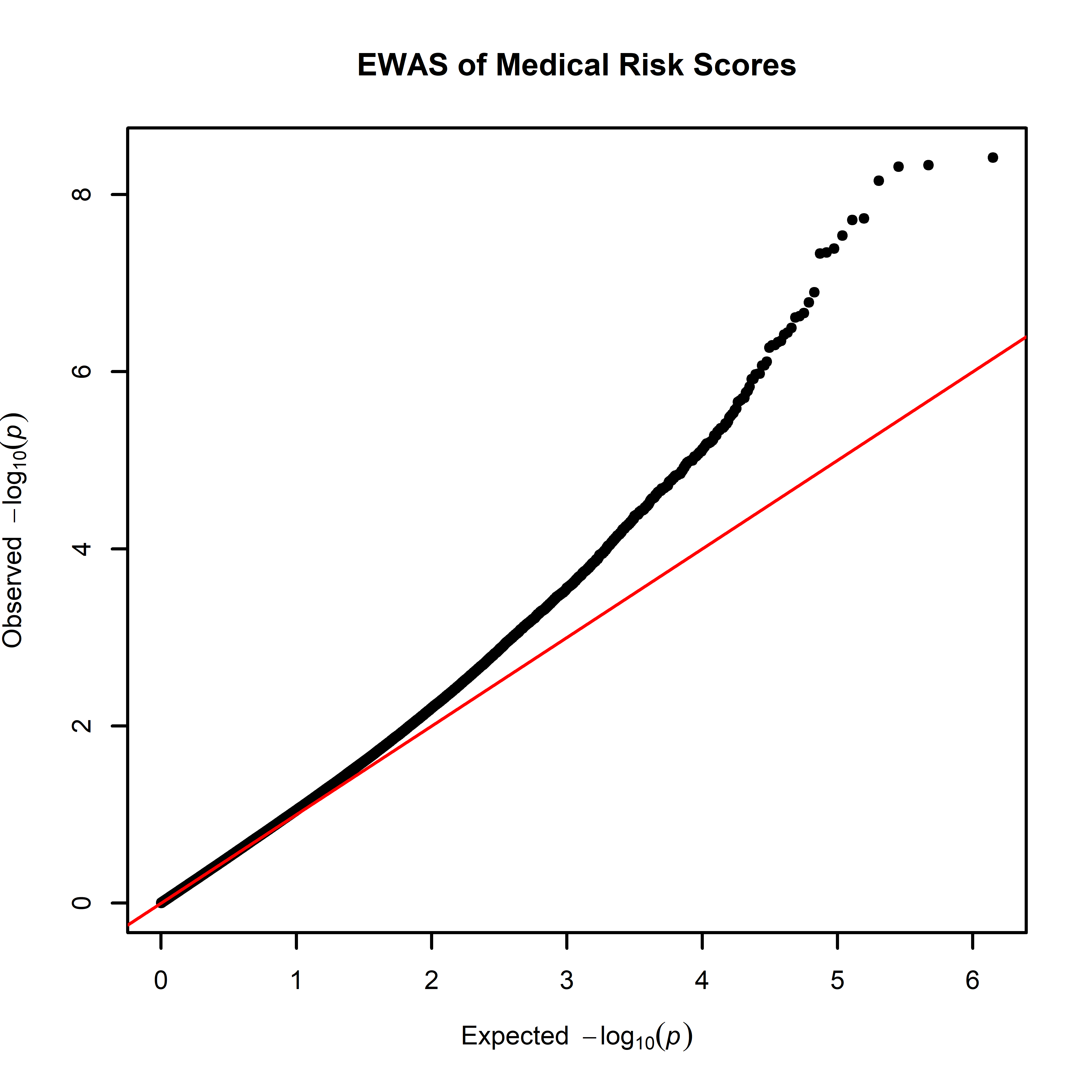


**Supplemental Figure S6:** Scatter plots of the magnitudes of the beta coefficients from the models in which DNAm was regressed on a 4-level factor for the neonatal morbidity risk scores with zero as the referent. We compared the magnitudes of differential methylation associated with a risk score of 2 versus a risk score of 1 (A), associated with a risk score of 3 versus a risk score of 1 (B), and associated with a risk score of 3 versus a risk score of 2 (C). Data points that rotate counter clockwise away from the diagonal line have a larger magnitude of effect for the risk score on the y-axis and are represented by blue dots while yellow dots represent those that had smaller magnitude of effect. Those with red circles around them represent the 10 CpGs that were significant after Bonferroni-correction from the EWAS.


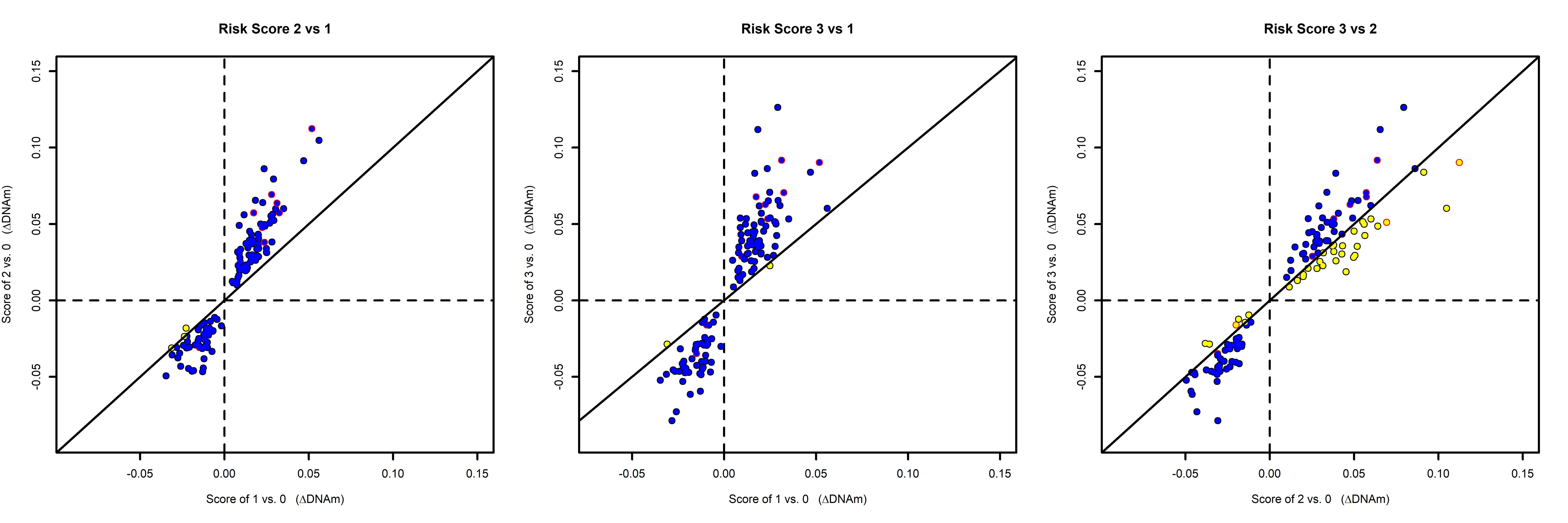


**Supplemental Figure S7:** Venn Diagram showing the overlap in the CpGs that were significantly associated with BPD, SBI, INF, and ROP among the 125 CpGs that were associated with the neonatal morbidity risk score in the EWAS at an FDR of 10%.

**
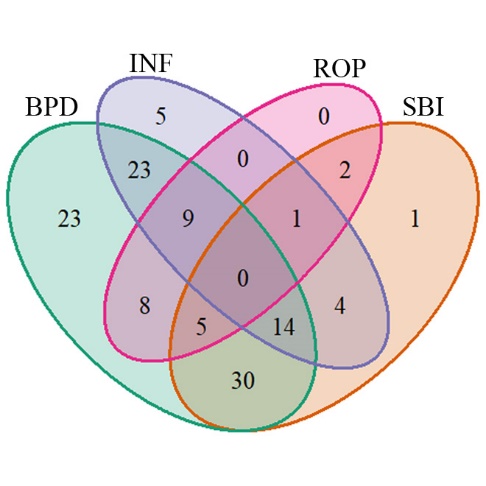
**

**Supplemental Figure S8:** The magnitudes of differential methylation associated with individual neonatal complication (A. BPD, B. SBI, C. INF, and D. ROP) were plotted against the magnitudes of differential methylation associated with an increase of one for the risk score for the 125 CpGs that were associated with the risk score in the EWAS.


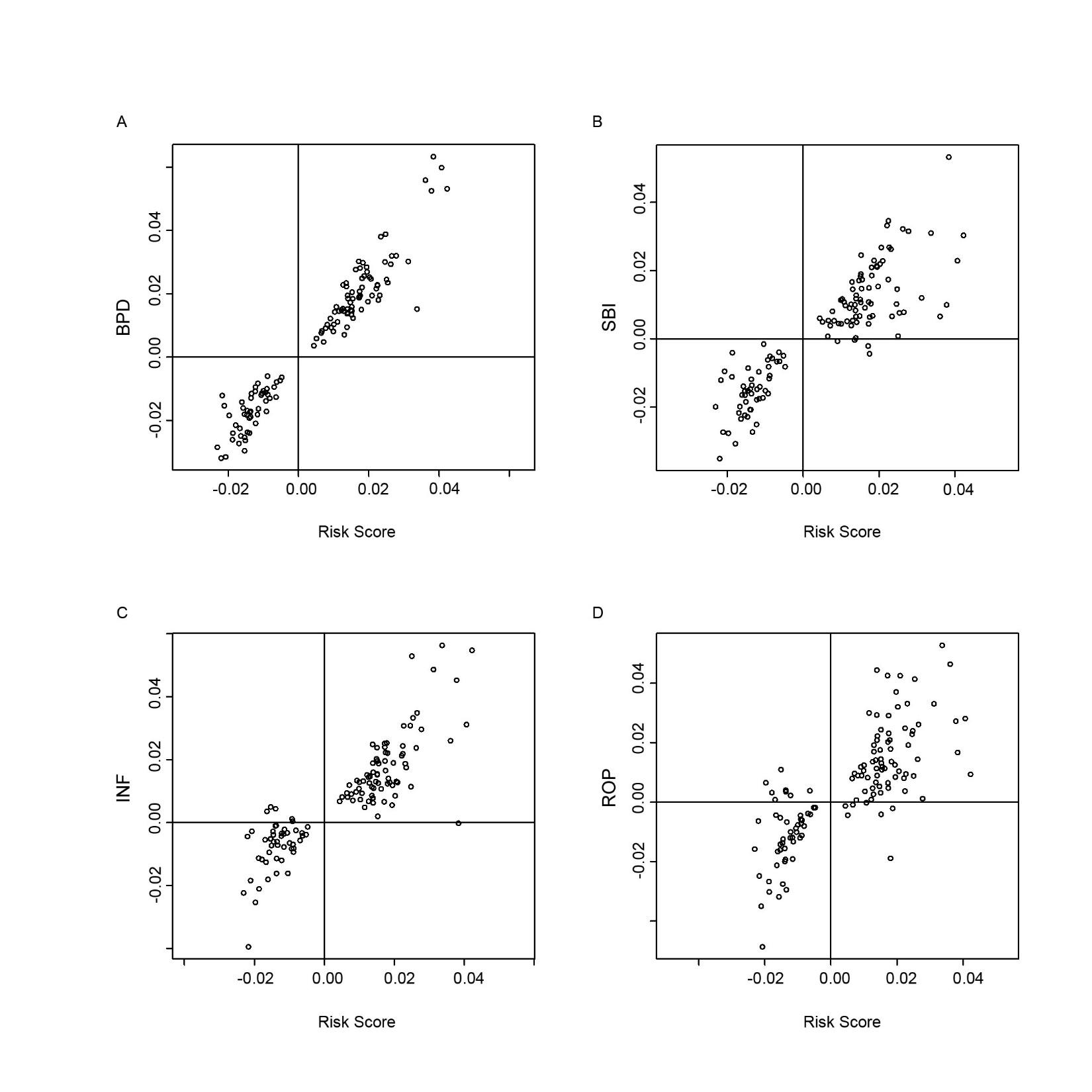

Supplement: Supplementary file 1 — Additional file 1: Supplemental Figures S1–S8. [file 13148_2020_942_MOESM1_ESM.docx]
